# RUCova: Removal of Unwanted Covariance in mass cytometry data

**DOI:** 10.1093/bioinformatics/btae669

**Published:** 2024-11-23

**Authors:** Rosario Astaburuaga-García, Thomas Sell, Samet Mutlu, Anja Sieber, Kirsten Lauber, Nils Blüthgen

**Affiliations:** Institute of Pathology, Charité-Universitätsmedizin Berlin, Berlin, 10117, Germany; Institute of Biology, Humboldt Universität zu Berlin, Berlin, 10117, Germany; Institute of Pathology, Charité-Universitätsmedizin Berlin, Berlin, 10117, Germany; Institute of Biology, Humboldt Universität zu Berlin, Berlin, 10117, Germany; Department of Radiation Oncology, University Hospital, LMU München, Munich, 81377, Germany; German Cancer Consortium (DKTK), Munich, 81377, Germany; German Cancer Research Center (DKFZ), Heidelberg, 69120, Germany; Institute of Pathology, Charité-Universitätsmedizin Berlin, Berlin, 10117, Germany; Institute of Biology, Humboldt Universität zu Berlin, Berlin, 10117, Germany; Department of Radiation Oncology, University Hospital, LMU München, Munich, 81377, Germany; German Cancer Consortium (DKTK), Munich, 81377, Germany; Clinical Cooperation Group ‘Personalized Radiotherapy in Head and Neck Cancer’ Helmholtz Center Munich, German Research Center for Environmental Health GmbH, Neuherberg, 85764, Germany; Institute of Pathology, Charité-Universitätsmedizin Berlin, Berlin, 10117, Germany; Institute of Biology, Humboldt Universität zu Berlin, Berlin, 10117, Germany; German Cancer Consortium (DKTK), Berlin, 10117, Germany

## Abstract

**Motivation:**

High dimensional single-cell mass cytometry data are confounded by unwanted covariance due to variations in cell size and staining efficiency, making analysis, and interpretation challenging.

**Results:**

We present RUCova, a novel method designed to address confounding factors in mass cytometry data. RUCova removes unwanted covariance from measured markers applying multivariate linear regression based on surrogates of sources of unwanted covariance (SUCs) and principal component analysis (PCA). We exemplify the use of RUCova and show that it effectively removes unwanted covariance while preserving genuine biological signals. Our results demonstrate the efficacy of RUCova in elucidating complex data patterns, facilitating the identification of activated signalling pathways, and improving the classification of important cell populations such as apoptotic cells. By providing a robust framework for data normalization and interpretation, RUCova enhances the accuracy and reliability of mass cytometry analyses, contributing to advances in our understanding of cellular biology and disease mechanisms.

**Availability and implementation:**

The R package is available on https://github.com/molsysbio/RUCova. Detailed documentation, data, and the code required to reproduce the results are available on https://doi.org/10.5281/zenodo.10913464.

## 1 Introduction

Mass cytometry allows for the simultaneous quantification of numerous cellular markers in individual cells and in multiple samples. It is widely used in immunology research to quantify surface proteins and classify immune cells (Bendall *et al.* 2011, Horowitz *et al.* 2013, Giesen *et al.* 2014, Spitzer and Nolan 2016, Georg *et al.* 2022). Mass cytometry is also increasingly used to study intracellular signalling pathways by measuring the abundance of phospho-proteins, providing information on various cellular processes such as colorectal cancer differentiation pathways (Brandt *et al.* 2019, Sell *et al.* 2023), organoid heterogeneity (Sufi *et al.* 2021), acute myeloid leukaemia (Han *et al.* 2015), and prediction of drug sensitivity in breast cancer (Tognetti *et al.* 2021). Although surface protein distributions typically show a bimodal pattern, those of intracellular signalling markers show an unimodal distribution with rather small quantitative shifts in response to perturbations. These distributions are affected by both biological and technical variability. Biological variability arises from inherent differences between individual cells, including variations in cell state, type, and size. In contrast, technical variability arises from experimental procedures and instrumentation, such as heterogeneous staining efficiency. While some biological variability is essential, unwanted variability, such as that caused by differences in cell size, carries the risk of confounding the data. This unwanted covariance can obscure the detection of small cell populations and prevent accurate comparisons between different experimental conditions, cell lines, and cell states.

In recent years, a class of methods under the umbrella term ‘remove unwanted variation (RUV)’ has been developed to address primarily the variation coming from batch effects. These methods have been successfully applied to various high-throughput data types, including microarrays (Gagnon-Bartsch and Speed 2012), RNA sequencing [RUV by Risso *et al.* (2014)], Nanostring nCounter gene expression [RUV-III by Molania *et al.* (2019)], single-cell RNA sequencing [scMerge by Lin *et al.* (2019)], as well as mass cytometry [CytofRUV by Trussart *et al.* (2020)]. Although differences between batches are a significant source of unwanted variability, single-cell mass cytometry datasets can exhibit considerable covariance within a single batch due to uncorrected heterogeneity in cell size and staining efficiency. Mass cytometry builds on flow cytometry by increasing the number of measurable markers because the mass spectrum is more specific than the fluorescence spectrum, which suffers from spectral overlap. In flow cytometry, marker abundance can be normalized by cell size using the Forward Scatter (FSC) parameter, which is proportional to the relative size of the cell. However, mass cytometry lacks an intrinsic parameter that serves directly as a proxy for cell size. Conventional normalization methods, such as those used in single-cell RNA sequencing, are impractical because of the lack of information on total protein content in mass cytometry data. Previous attempts to normalize cell volume using Ruthenium isotopes (Rapsomaniki *et al.* 2018) faced challenges with complexity and assumptions about the relationship between marker and cell volume. To address these limitations, we introduce RUCova, a novel approach that uses linear modelling based on surrogates of sources of unwanted covariance (SUCs). This approach is based on the assumption that global protein abundance is confounded by SUC, such as cell size and staining efficiency. By incorporating factors such as mean DNA, mean barcoding isotopes, pan Akt, and total ERK, RUCova effectively removes technical artefacts and improves the precision of mass cytometry analyses. Our study demonstrates the utility of RUCova in revealing complex data patterns, identifying activated signalling pathways, and improving the classification of important cell populations, such as apoptotic cells. In this article, we present the unique advantages of RUCova in advancing our understanding of cellular biology and disease mechanisms.

## 2 Materials and Methods

RUCova comprises two major steps. First, it fits a multivariate model for each measured marker (m) across cells (i) from samples ($j_{i}$) with respect to the surrogates of SUC $\vec{x_{i}}$. Second, it eliminates such dependency by assigning the residuals ϵ of the model as the new modified expression of the marker. The fit can be expressed as:

(1) $$y_{i}^{m}({\vec{x}}_{i},j_{i})=\underset{M({\vec{x}}_{i},j_{i})}{\underset{︸}{O^{m}(j_{i})+S^{m}({\vec{x}}_{i},j_{i})}}+\epsilon_{i}^{m}$$

where $S^{m}({\vec{x}}_{i},j_{i})$ describes the slope of the fit and $O^{m}(j_{i})$ the intercept or offset. The predictors are SUCs $\vec{x_{i}}$, which can be specific markers as proxies of the confounding factors or the principal components (PCs) derived from a principal component analysis (PCA) performed on such proxy markers.

**Figure 1.**
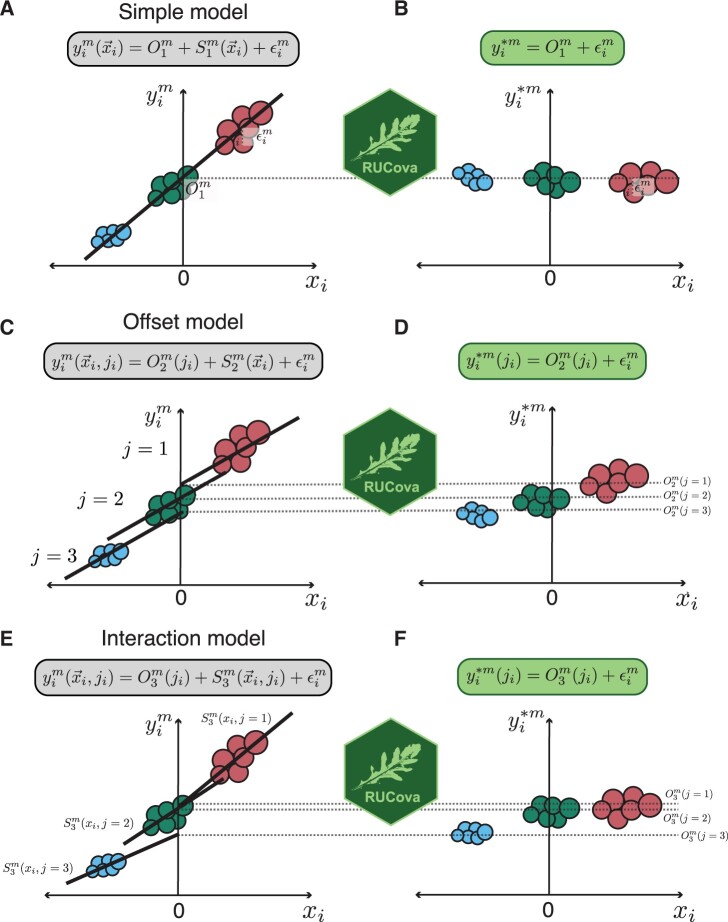
Illustration of the RUCova method on a fictional dataset of 3 samples (in three different colours), and its three different models. (A, C, E) Original expression $y_{i}^{m}$ of a marker *m* before RUCova as a function of a centred expression of a SUC or PC. Illustrative regression line and equation corresponding to each model (in boxes). (B, D, F) Modified expression $y_{i}^{*m}$ of a marker *m* after applying RUCova. (A, B) Simple model: one fit across the input dataset with intercept $O_{1}^{m}$ and residuals $\epsilon_{i}$. (C, D) Offset model: one slope $S_{2}^{m}({\vec{x}}_{i})$ for the whole input dataset and different intercepts $O_{2}^{m}(j_{i})$ between samples. (E, F) Interaction model: one fit per sample *j* with intercepts $O_{3}^{m}(j_{i})$ and slope $S_{3}^{m}({\vec{x}}_{i},j_{i})$. (D, F) Keeping the offset $O^{m}(j_{i})$ between samples *j* after applying RUCova.

RUCova offers three different uni- or multivariate linear models to describe the relationship between marker expression and SUC: (1) $M_{1}({\vec{x}}_{i})$: simple, (2) $M_{2}({\vec{x}}_{i},j_{i})$: offset, and (3) $M_{3}({\vec{x}}_{i},j_{i})$: interaction (Fig. 1).

1. **Simple model (** Fig. 1A and B **):** Consists of a fit for the measured intensity values $y_{i}^{m}$ for each marker m throughout the dataset.
  (2) $$M_{1}({\vec{x}}_{i})=\underset{=O_{1}^{m}}{\underset{︸}{\beta_{0}^{m}}}+\underset{=S_{1}^{m}({\vec{x}}_{i})}{\underset{︸}{\sum_{p=1}^{N_{\mathrm{SUC}}}\alpha_{p}^{m}\cdot x_{i,p}}}$$
  where $\beta_{0}^{m}$ is the intercept and $\alpha_{p}^{m}$ is the slope coefficient for each marker m and predictor or SUC p.
2. **Offset model (** Fig. 1C and D **):** Consists of a fit for the measured intensity values $y_{i}^{m}$ for each marker m and sample $j_{i}$. The fits for the samples share the same slope, while differing in the intercept (offset term $O_{2}^{m}(j_{i})$).
  (3) $$M_{2}({\vec{x}}_{i},j_{i})=\underset{=O_{2}^{m}(j_{i})}{\underset{︸}{\beta_{j_{i}}^{m}}}+\underset{=S_{2}^{m}({\vec{x}}_{i})}{\underset{︸}{\sum_{p=1}^{N_{\mathrm{SUC}}}\alpha_{p}^{m}\cdot x_{i,p}}}.$$
3. **Interaction model (** Fig. 1E and F **):** or the measured intensity values $y_{i}^{m}$ for each marker m and sample $j_{i}$. The fits for the samples can have different slopes (interaction term $S_{3}^{m}(\vec{x_{i}},j_{i})$) and intercepts (offset term $O_{3}^{m}(j_{i})$).
  (4) $$M_{3}({\vec{x}}_{i},j_{i})=\underset{=O_{3}^{m}(j_{i})}{\underset{︸}{\beta_{j_{i}}^{m}}}+\underset{=S_{3}^{m}({\vec{x}}_{i},j_{i})}{\underset{︸}{\sum_{p=1}^{N_{\mathrm{SUC}}}\alpha_{j_{i},p}^{m}\cdot x_{i,p}}}.$$

Samples $j_{i}$ can be different cell lines, perturbations, conditions, *metacells* (clusters), or even batches. By taking the zero-centered distributions of the SUCs ($x_{i}^{c}=x_{i}-\frac{1}{N_{i}}\sum_{i}^{N_{i}}x_{i}$), the mean values of the markers m across all cells are kept after applying RUCova (Fig. 1). If a more conservative approach is desired, where the log-fold changes between samples should be kept, each SUC should be centred per sample (Fig. 1). Similarly, when using PCs as the predictive variables, SUCs can be *z*-score normalized by the sample before performing PCA.

The RUCova method eliminates the dependency of each measured marker on the SUCs by computing the model’s residuals and the intercept as the revised expression for each marker ($y_{i}^{m*}$). The offset—intercept—term $O^{m}$ for different samples can be wanted or unwanted. For the first case, the new and modified abundance of the marker after applying RUCova is independent of ${\vec{x}}_{i}$ and can be expressed as:

(5) $$y_{i}^{*m}(j_{i})=O^{m}(j_{i})+\epsilon_{i}^{m},$$

where $\epsilon_{i}^{m}$ are the residuals of the model.

More information about the RUCova model, cell cultures and mass cytometry measurements can be found in the Supplementary Material.

## 3 Results

### 3.1 The mass cytometry data are confounded by multiple factors

Mass cytometry enables the quantification of protein and phospho-protein abundance in single cells using antibodies conjugated with metal isotopes, facilitating the investigation of intracellular signals that determine the state and response to treatments. However, challenges such as heterogeneous cell volume and labelling efficiency confound the data, leading to spurious correlations between markers and hindering comparisons between cell lines, perturbations, and cell states. To address this, we developed RUCova, an R package designed to remove unwanted covariance in mass cytometry data.

**Figure 2.**
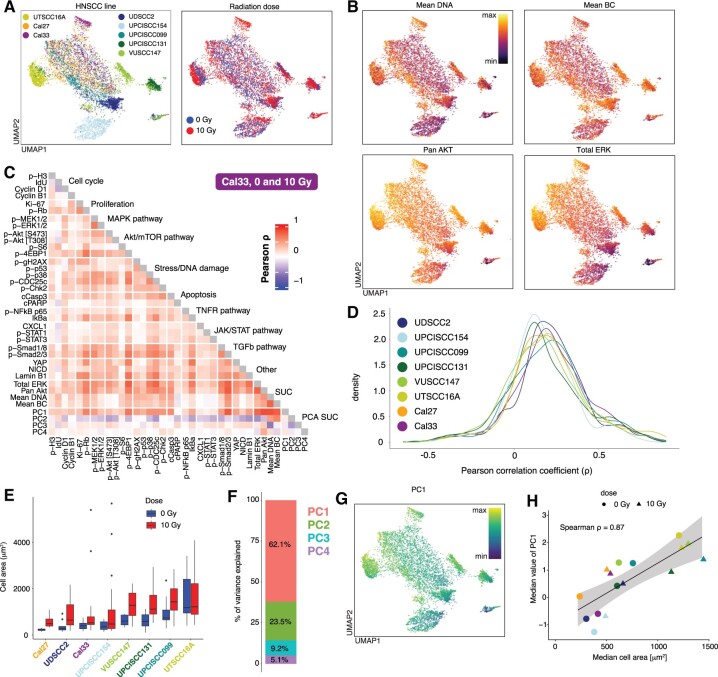
Unwanted covariance in a mass cytometry dataset coming from heterogeneous cell area and other factors. (A) UMAP coloured by cell line (left) and dose (right) calculated by excluding cell cycle and proliferation markers (*n* = 500 cells per line and dose). (B). UMAP is coloured by the expression values of the 4 SUCs: mean DNA (top left), mean BC (top right), pan Akt (bottom left), and total ERK (bottom right). Expression values were asinh-transformed and min-max normalized. (C). Pearson correlation coefficients between asinh-transformed and *z*-scored expression values of markers in the Cal33 cell line across cells from 0 and 10 Gy. (D). Distribution of Pearson correlation coefficients between markers in all cell lines across cells from 0 and 10 Gy. (E) Measurements of the cell area via microscopy images before fixation (*n* = 16.5 ± 3.7 cells per cell line and condition, Supplementary Fig. S6B). (F). Percentage of variance explained by each PC of a PCA based on the SUCs. PCA was calculated based on the asinh-transformed and *z*-scored expression values of the four SUCs in (B). (G). UMAP coloured by PC1 of a PCA based on the 4 SUCs. PC1 values were min-max normalized. (H) Spearman correlation coefficients between the median cell area (${\mu m}^{2}$) from (E) and median value of PC1 from (G) per cell line and dose.

To illustrate the need and benefit of using the RUCova method, we chose a mass cytometry dataset with eight different head-and-neck squamous cell carcinoma (HNSCC) cell lines in control (0 Gy) and irradiated (48 h after 10 Gy) condition (Fig. 2A). Since cell volume and labelling efficiency cannot be directly measured with mass cytometry, we use four surrogates of SUCs (Supplementary Equation S1 and Fig. S2A and Fig. 2B) which strongly correlated with each other and with the majority of markers in all studied HNSCC cell lines (Supplementary Figs S3 and S4): (i) mean DNA: is the mean value of normalized iridium channels (Supplementary Equation S2). Iridium is a common DNA stain in mass cytometry. Interestingly, we noted that DNA staining was highly correlated with ruthenium staining (Supplementary Fig. S5A–C), previously proposed by Rapsomaniki *et al.* (2018) to measure cell volume. (ii) Mean BC: mean value of the highest (used) barcoding isotopes per cell (Supplementary Equation S3). Mass cytometry is often performed with multiplexed samples that are stained with a specific combination of isotopes, e.g.: palladiums or telluriums (Zunder *et al.* 2015, Willis *et al.* 2018), acting as a barcode. These barcoding reagents bind unspecifically to surface proteins, but also to intracellular proteins when stained after cell fixation (Zunder *et al.* 2015). Hence, barcode signals might be used as a surrogate of cell volume. (iii) pan Akt, and (iv) total ERK. Total ERK and pan Akt are commonly used as loading controls for normalization in e.g.: western blotting experiments, as they are typically abundant proteins that are relatively stable under different experimental conditions. The correlations between the marker’s signals for the Cal33 cell line are depicted in Fig. 2C as an example. Some correlations are authentic and expected, such as between proliferation markers (p-Rb and Ki-67), members of the MAPK pathway (p-MEK1/2 and p-ERK1/2), regulators of cell cycle progression and protein synthesis (p-Rb and p-4EBP1), proteins in the DNA damage response pathway (p-p53 and p-γH2AX), and proteins involved in apoptotic cell death (p-Chk2, cCasp3, cPARP, NF-κB, and IκBα). However, other correlations are suspicious and most likely driven by unwanted covariance, especially between p-Rb, p-38, p-CDC25c, p-Smads, YAP, and the majority of the measured markers (Fig. 2C). This distribution of correlation coefficients is observed in all the studied HNSCC lines (Fig. 2D). We quantified the cell area via microscopy images for each HNSCC cell line and condition (Fig. 2E and Supplementary Fig. S6A and B). We observed variations in the median cell area, both across different cell lines within the same condition and for each cell line across radiation conditions. The median area of the cells increased after irradiation, which agrees with previous observations (Rene and Nardone 1968, Ronny Sham *et al.* 2020, Ren *et al.* 2023). The UPCISCC131 cell line showed the highest increase in the median cell area after irradiation. The PCA conducted across the four SUCs revealed that 62% of the variance of the SUCs is captured by PC1 (Fig. 2F and G). Interestingly, PC1 showed a strong correlation with the measured median cell area (ρ = 0.87, 2 H), while the subsequent PCs did not show such a correlation (Supplementary Fig. S6D). This suggests that cell size is likely the primary SUC in this dataset. Furthermore, all SUCs had positive and similar loading values for PC1 (Supplementary Fig. S2B), indicating that cell size could uniformly influence both antibody-based proxies (such as total ERK and pan Akt) and non-antibody-based proxies (such as mean DNA and BC). To further support this hypothesis, we tested the ASCQ-ruthenium (Ru) compound [proposed by Rapsomaniki *et al.* (2018) to correlate with cell volume] on the unperturbed Cal33 cell line (Supplementary Fig. S5). In this analysis, PC1 across pan Akt, total ERK, mean DNA, and mean Ru accounted for 64% of the variance (Supplementary Fig. S5C) with all SUCs contributing similarly to PC1 (Supplementary Fig. S5D). In both datasets, PC2 was primarily influenced by the antibody-based proxies pan Akt and total ERK (Supplementary Figs S2B and S5D) likely representing staining efficiency as a secondary SUC.

In an uncorrected mass cytometry dataset, these differences in cell area (and therefore marker abundance) and other factors, such as staining efficiency, will confound the comparisons between cell lines and perturbations, masking meaningful biological information.

### 3.2 RUCova enables an improved classification of apoptotic cells by uncovering previously obscured cell populations

To address unwanted covariance mainly due to heterogeneous cell area and staining efficiency, we applied the RUCova method using the interaction model $M_{3}({\vec{x}}_{i},j_{i})$ with cell lines treated as separate samples (j). This approach enabled us to account for the possibility that different cell lines may not have exhibited the same relationship between protein abundance and SUCs, such as cell size or staining efficiency and ensured that perturbation analysis was not confounded by radiation-induced increases in cell area by applying one fit across irradiated and non-irradiated cells.

**Figure 3.**
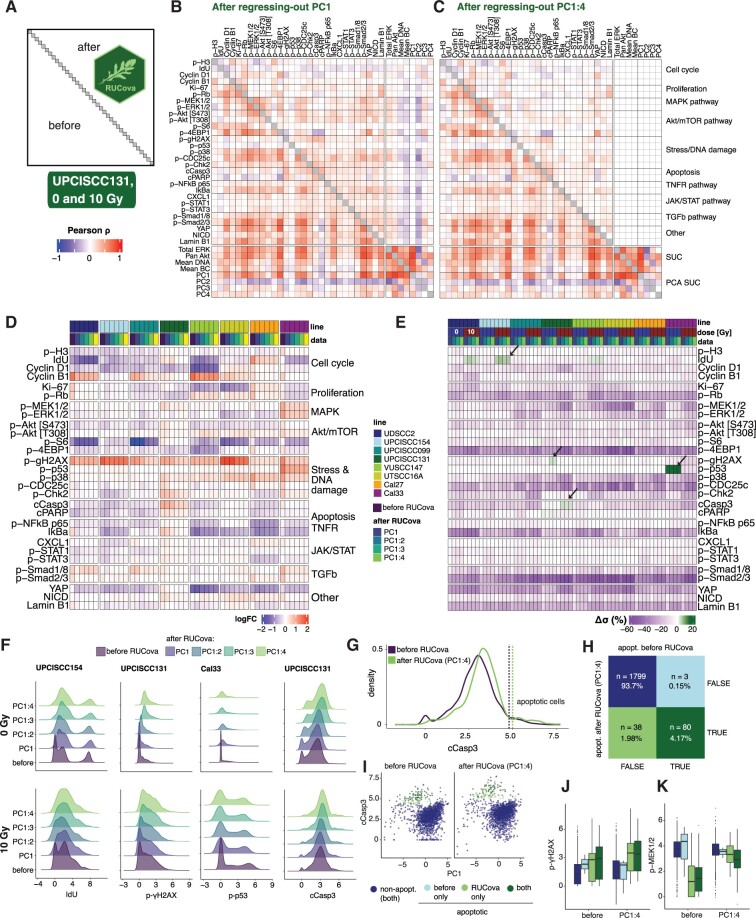
RUCova removes unwanted covariance and allows improved classification of apoptotic cells. (A) Scheme of the correlation heatmap, where the lower and upper triangles show the Pearson correlation coefficients between marker values before and after RUCova (using the interaction model $M_{3}({\vec{x}}_{i},j_{i})$ per cell line), respectively. Diagonal unity values are depicted in grey. Pearson correlation coefficients were calculated between the asinh-transformed and z-scored expression values of markers. (B, C) Correlation heatmap with the upper triangle showing the Pearson correlation coefficients between marker values in the UPCISCC131 cell line across cells from 0 and 10 Gy after applying RUCova based on (B) PC1, (C) PC1–PC4. (D) Heatmap of fold changes of asinh-transformed data of measured markers between irradiated and non-irradiated condition for each cell line, and dataset (before or after applying RUCova’s interaction model). (E) Heatmap of percentual difference in the standard deviation σ of each marker’s distribution after applying RUCova relative to the distributions before RUCova ($\Delta \sigma =\frac{\sigma_{\text{RUCova}}-\sigma_{\text{before}}}{\sigma_{\text{before}}}\cdot 100$) per cell line and dose levels. Arrows indicate specific cases where Δσ>0. (F) Density plots for asinh-transformed expression values of IdU, p-γH2AX, p-p53, and cleaved Casp3 before and after RUCova (*y*-axis) in cell lines where Δσ>0 (arrows in panel E) for 0 Gy (first row) and 10 Gy (second row). (G) Density plot for asinh-transformed expression of cleaved Casp3 in the irradiated (10 Gy) UPCISCC131 cell line, before and after RUCova based on all four PCs. Dashed vertical lines indicate the decision thresholds for apoptotic (higher values of cleaved Casp3) and non-apoptotic populations according to the cleaved Casp3 distribution before and after applying RUCova. (H) Confusion matrix for classification of apoptotic cells in the irradiated UPCISCC131 cell line before and after RUCova (based on all four PCs) according to the decision thresholds in (G). (I) Scatter plots of PC1 vs. asinh-transformed values of cleaved Caspase-3 before (left) and after RUCova (right) for irradiated cells in the UPCISCC131 line. Cells are coloured by apoptotic status. (J, K) Boxplots of asinh-transformed expression of (J) p-γH2AX and (K) p-MEK1/2 before and after RUCova in irradiated UPCISCC131 cells according to their apoptotic status.

After removing the covariance with all SUCs (i.e., using all four PCs as predictive variables) spurious correlations were removed and authentic correlations were kept (Fig. 3A–C), enabling differentiation between activated (apoptotic and MAPK pathway), and non-activated signalling pathways (JAK/STAT and TGFβ). The correlations between marker abundances were substantially decreased in all HNSCC cell lines, especially after regressing out the correlation with PC1 and PC2 (Supplementary Figs S3 and S4). While RUCova does correct for confounding factors, it preserves the key treatment effects like increased phosphorylation of p-γH2AX, and line-specific treatment effects like phosphorylation of p-p53 and activation of MAPK pathway in the Cal33 cell line (Fig. 3D).

As expected when removing unwanted covariance, we observed a general decrease in the standard deviation (σ) of the signals for each condition after applying RUCova (Fig. 3E). However, for some markers, we observed an unexpected increase in standard deviation relative to the original distributions (arrows in Fig. 3E). For IdU in the UPCISCC154 line, p-γH2AX in the UPCISCC131 line, and p-p53 in the Cal33 line, the higher standard deviation observed after RUCova was attributed to the assignment of non-zero values to the artificial zero values typically present in a mass cytometry measurement (Fig. 3F). The rise in the standard deviation of the distribution of the apoptotic marker cleaved Caspase-3 (cCasp3) in the irradiated UPCISCC131 line can be attributed to two factors: the assignment of nonzero values and an increased dissimilarity in the signal of cCasp3 between the non-apoptotic and apoptotic populations (Fig. 3F and G). We categorized the apoptotic cells in the irradiated UPCISCC131 cell line by analyzing their cCasp3 signals and establishing decision thresholds before and after RUCova (regressing-out on PC1 to PC4) (dashed vertical lines in Fig. 3G). The classification based on the data after applying RUCova allowed us to increase the identification capabilities of apoptotic cells by 47.5% (Fig. 3H). Before applying RUCova, apoptotic cells showed lower original cCasp3 signals, making them less distinguishable from nonapoptotic cells. This originally lower cCasp3 signal corresponded to lower PC1 values (Fig. 3I). After RUCova, the expected differences in markers like the DNA-damage marker p-γH2AX between apoptotic and non-apoptotic cells became discernible (Fig. 3J and Supplementary Fig. S7), while potential artefacts, like lower p-MEK1/2 signals in apoptotic cells, were reduced (Fig. 3K and Supplementary Fig. S7).

To evaluate RUCova’s performance in recovering ground truth correlations amidst introduced artefacts, we conducted a validation study by simulating 100 ground-truth mass cytometry datasets using the CytoGLMM R package [Seiler *et al.* (2021), Supplementary Fig. S8]. Artefacts were introduced in the form of linear and nonlinear (quadratic) modifications to marker values based on artificial cell size values, which was drawn from a log-normal distribution. The results demonstrate that RUCova effectively restores original correlations and condition-specific patterns, achieving precision and recall values close to 1.0 in the task of identifying significant markers between treatment and control conditions.

The use of RUCova in the HNSCC dataset enabled the preservation of authentic correlations while removing spurious ones, facilitating the differentiation between activated and non-activated signalling pathways after irradiation in different HNSCC lines. The application of RUCova allowed a clearer understanding of apoptotic marker distribution and its relation to cell size reduction, highlighting its efficacy in elucidating complex data patterns in mass cytometry analysis.

### 3.3 RUCova enhances the reliability of perturbation comparisons by eliminating cell size artefacts

**Figure 4.**
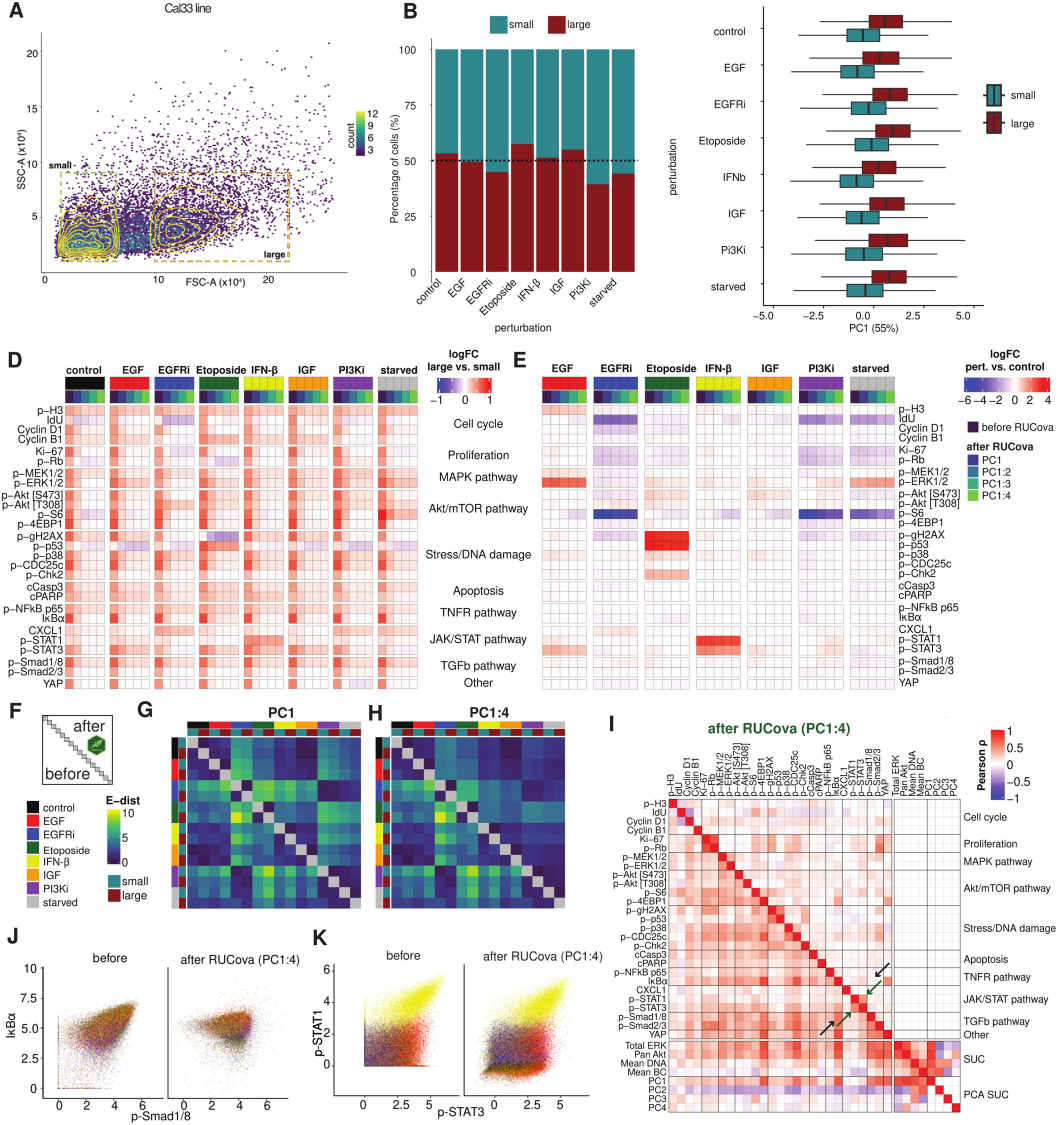
RUCova successfully removes cell size artefacts enabling accurate perturbation analyses. (A) Density scatter plot of the FACS parameters SSC-A vs. FSC-A. Cells are binned and coloured by counts. Gates for size-sorted groups of small and large cells in the Cal33 cell line before mass cytometry measurement. (B) Percentage of small and large cells per perturbation condition in the Cal33 cell line. (C) Boxplots of PC1 (of a PCA based on the four SUCs) per perturbation and size group. (D) Heatmap of fold-change (FC) of asinh-transformed values between large and small cells in all conditions, before and after applying the simple RUCova model ($M_{1}({\vec{x}}_{i})$) using different numbers of PCs. (E) Heatmap of fold-change (FC) of asinh-transformed values between perturbation and control condition, before and after applying the simple RUCova model ($M_{1}({\vec{x}}_{i})$ using different number of PCs. (F) Scheme of the E-distance heatmap, where the lower and upper triangles show the E-distance between conditions before and after RUCova (using the simple model $M_{1}({\vec{x}}_{i})$), respectively. (G, H) E-distance heatmap between 1000 cells per condition and sorted population for data after applying RUCova using (G) only PC1, (H) PC1–PC4. (I) Correlation heatmap with the upper triangle showing the Pearson correlation coefficients between marker values across all perturbations and sorted populations after RUCova based on PC1 to PC4. Black arrows indicate an artefactual correlation which is removed after applying RUCova, and green arrows indicate a real correlation which is kept. (J, K) Scatter plots of Cal33 cells coloured by perturbation before (left) and after (right) applying RUCova based on all four PCs. (J) asinh-transformed values of p-Smad1/8 vs. IκBα (artefactual correlation). (K) asinh-transformed values of p-Stat1 and p-Stat3 (real correlation driven by IFN-β-stimulated cells).

To understand how cell size and other factors can confound mass cytometry data and especially analyses of cellular responses, we perturbed cells from the HNSCC Cal33 cell line using EGF stimulation (30 min), EGFR inhibition (Gefitinib, 24 h), Etoposide treatment (2 h), IFN-β stimulation (30 min), IGF stimulation (30 min), PI3K inhibition (24 h), and starvation alone (24 h). We then sorted the cells into two groups based on size (small and large cells) using fluorescence-activated cell sorting (FACS) (Fig. 4A).

Overall, most of the perturbations resulted in similar proportions of sorted cells, as shown in Fig. 4B. However, in the case of PI3K inhibition, most of the sorted cells were smaller. The PC1 of a PCA based on SUCs was substantially higher for large cells compared to small cells (Fig. 4C). Correspondingly, average marker values were consistently higher in large cells compared to small cells across all perturbations (Fig. 4D), illustrating once again how cell size confounds mass cytometry data. In this dataset, we utilized the simple model $M_{1}({\vec{x}}_{i})$, characterized by a single slope and intercept per marker, ideal for examining a single cell line. This method facilitates the comparison of different perturbations by considering potential confounders, such as variations in cell size between perturbations, to ensure that they do not obscure the true effects of the perturbations. Upon applying RUCova, differences in marker mean values between large and small cells were notably reduced, particularly after removing correlations with all four PCs. For certain markers and perturbations (e.g., p-p53 in Etoposide treatment and p-STAT1 in IFN-β stimulation) considerable fold changes between large and small cells persisted after RUCova, indicating the method’s ability to preserve genuine biological signals while eliminating artificial ones. Across large and small cells, the fold changes between perturbation and control conditions were usually maintained after applying RUCova (Fig. 4E). However, the removal of unwanted covariance led to modified fold-changes in some cases: p-ERK1/2 increased after PI3K inhibition relative to control, which could indicate a compensatory signalling mechanism as a means of maintaining cell survival and proliferation. These reductions or increments in the fold-change after RUCova could also be due to the unbalanced proportion of small and large cells in a perturbation compared to the control condition. By assessing the pairwise E-distance (Peidli *et al.* (2024)) between perturbation conditions and sorted populations both before and after applying RUCova, we observed that differences between small and large cells were effectively eliminated, along with dissimilarities arising from variations in cell size distribution, such as those seen in the PI3K condition compared to the others. We also observed small increments in the E-distance after implementing RUCova, especially between EGFR inhibition and starvation (Fig. 4F–H and Supplementary Fig. S9A).

The Pearson correlation coefficients between markers across the entire dataset after RUCova provide reliable information. An example of a regressed-out artefactual correlation is p-Smad1/8 and IκBα (black arrow in Fig. 4I and J), which was mainly driven by the size of the cells (Supplementary Fig. S9C). An example of a real correlation that was kept after RUCova is between p-Stat1 and p-Stat2 mainly driven by IFN-β stimulation (green arrow Fig. 4I and K and Supplementary Fig. S9D).

This suggests that RUCova successfully reduced the dissimilarities between conditions due to cell size and other potential SUC, providing a clearer understanding of the underlying biological responses to different stimuli.

## 4 Discussion

Mass cytometry data are contaminated by variance that is induced by heterogeneous cell size, staining efficiency, and other technical artefacts that lead to spurious correlations between markers. Here we describe RUCova, a method to regress out such unwanted co-variation. The method consists of fitting a model for each marker based on surrogates of SUCs, such as mean DNA, mean barcode signal, and total protein markers such as total ERK and AKT. Previous approaches used a fixed relation between the abundance of all markers and cell size stains (Rapsomaniki *et al.* 2018), producing suboptimal results, as the extent of correlation between protein abundance and cell size varies and depends on, e.g. protein localization (Lanz *et al.* 2023).

Cell size can exhibit variability across different cell lines, cell types (e.g. various immune cells), and even within different tissue microenvironments (Liu *et al.* 2022). If the relationship between cell size and protein abundance varies between samples, RUCova can incorporate this using the interaction model ($M_{3}(\vec{x_{i}},j)$), where the slopes are specific for each sample (cell lines, cell types, or cell clusters).

In Fig. 2 and Supplementary Figs S3 and S4 we illustrated that the Pearson correlation coefficients between markers display consistently high values, posing challenges in distinguishing between activated and nonactivated signalling pathways. Most of these markers exhibited strong correlations with SUCs, especially with the first principal component (PC1), which explained 62% of the variance of the SUCs and were highly correlated with cell area (Fig. 2H), thereby confirming the artefactual nature of these elevated correlations.

In this study, the existing SUCs may not fully separate cell size from staining efficiency since both influence marker abundance similarly. However, PCA suggests that PC1 primarily captures cell size as the main SUC, affecting antibody-based and non-antibody-based SUCs, while PC2 likely reflects staining efficiency, mainly influencing antibody-based markers such as pan Akt and total ERK (Supplementary Fig. S2B and S5D). Developing new surrogates to disentangle these factors better is a promising area for future research. Our choice of antibody-based SUCs (total ERK and pan Akt) aligns with our marker’s panel and our focus on the MAPK pathway. However, alternatives like GAPDH could be useful in different contexts, given its frequent use as a control in experiments like western blots.

Figure 3 illustrates our effective mitigation of spurious correlations and variance in marker distributions using RUCova. By removing PC1 and PC2 through regression, we successfully eliminated these spurious correlations. For control over the removal of unwanted covariance, we advise using PCA components on the SUC as model predictors. With this, PC1 might serve as a proxy for cell size in future analyses.

Zero values in mass cytometry data often arise due to the instrument’s sensitivity limits. Some approaches developed for mass cytometry data address these zero values by imputing them with estimated values (Li *et al.* 2017, Minoura *et al.* 2021) or by extrapolating measurements from other panels using *k*-nearest neighbour methods (Abdelaal *et al.* 2019). However, applying imputation to uncorrected data can introduce bias, as the imputed values may be influenced by existing covariance. RUCova directly fits a linear model that includes the zeros and assigns non-zero values according to the removed unwanted covariance. This ensures that imputed values are assigned while removing the bias from such covariance.

An important benefit of applying RUCova is the identification of cell populations that may be obscured due to heterogeneous cell sizes. In this article, we showed that RUCova allowed us to identify about 50% more apoptotic cells in a dataset. These previously hidden cells exhibited the lowest PC1 values, suggesting that they were smaller in size, a known phenomenon in apoptotic cells that undergo cell shrinkage during the early stages of apoptosis (Kerr *et al.* 1972, Jänicke *et al.* 1998, Albeck *et al.* 2008). Therefore, we propose that a two-dimensional gating approach utilizing cCasp3 and PC1 could enhance the classification of apoptotic cells. Although a one-dimensional classification method based on cCasp3 was used, RUCova adjusted the marker expression values for apoptotic signals, such as increased levels of the DNA damage marker p-γH2AX, relative to nonapoptotic cells (Fig. 3J).

To directly compare how RUCova corrects the data between small and large cells, we employed FACS to separate large and small cells on a pool of perturbed and unperturbed cells from the Cal33 cell line (Fig. 4). RUCova removed differences in signals between small and large cells with only very few biologically plausible exceptions. These include p-Stat1 which showed differences between large and small cells following IFN-β stimulation. IFN-β induces inflammation which leads to larger cell sizes (Han *et al.* 2022), thus higher p-Stat1 signal may be a genuine signal indicative of inflammation-induced cell enlargement.

We carried out a validation study (Supplementary Fig. S8), demonstrating that RUCova can accurately reconstruct ground truth simulated biological signals despite various noise types (linear and non-linear) and strengths. Its consistent precision and recall in identifying significant changes in marker expression between conditions highlight its utility for mass cytometry, especially in intricate experimental scenarios.

It is important to select a suitable model based on experimental design and research aims. RUCova provides three tiers of linear models (simple, offset, and interaction) customized for various contexts. The simple model (Fig. 1A and B) is suitable for datasets with one cell line/organism/biological system and multiple perturbations to assess treatment effects. In the ligand/inhibition dataset (Fig. 4), fitting a simple model across perturbations while centring SUCs can eliminate artefacts. This model is advisable when the relationship between the marker abundance and the confounding factors (slope) is uniform across the dataset. The offset model is also appropriate when the dataset contains one biological system, but different intercepts between samples (e.g. batches) must be accounted for. It is beneficial for removing unwanted log-fold changes between samples, such as batch effects, while fitting a common slope across the data. The interaction model (Fig. 1E and F) is best for datasets involving different cell types or when comparing treatment effects between cell lines with different marker’s abundance relationships to unwanted covariance (e.g. cell size). In the HNSCC dataset (Figs 2 and 3), with interline cell size variation, an interaction model per line is advisable.

While RUCova aims to improve the interpretability and reliability of mass cytometry data by removing unwanted covariances, it is essential to consider the potential impact of data correction on biological interpretation. Overcorrection or removal of genuine biological signals alongside technical artefacts may obscure meaningful biological insights or introduce biases into downstream analyses. To ensure the correction preserves biological relevance, we suggest evaluating changes in key metrics, such as correlation coefficients between markers and fold-changes of (asinh- or log-) transformed data between conditions of interest. As demonstrated here, these metrics should be compared with previous biological knowledge to assess the impact of the correction.

## 5 Conclusion

In conclusion, our study introduces RUCova as a powerful tool for removing unwanted covariance in mass cytometry data, thereby enhancing the accuracy and reliability of downstream analyses. By effectively addressing technical artefacts associated with heterogeneous cell size and staining efficiency, RUCova facilitates the uncovering of genuine biological signals and contributes to a deeper understanding of cellular processes. Our findings demonstrate the utility of RUCova in elucidating complex data patterns, facilitating the identification of activated signalling pathways, and improving the classification of apoptotic cells. Furthermore, we emphasize the importance of thoughtful model selection and validation, as well as the critical interpretation of results in the context of biological insights. Moving forward, continued refinement and validation of RUCova and related methodologies will further enhance their utility in advancing our understanding of cellular biology and disease mechanisms.

## Supplementary Material

btae669_Supplementary_Data

## Data Availability

R package is available on https://github.com/molsysbio/RUCova. Detailed documentation, data, and the code required to reproduce the results are available on https://doi.org/10.5281/zenodo.10913464.
