# Supplementary material for: RUCova: Removal of Unwanted Covariance in mass cytometry data
Source: Bioinformatics. 2024 Nov 23;40(11):btae669. doi: 10.1093/bioinformatics/btae669 (PMC11601163; doi:10.1093/bioinformatics/btae669)
Supplement: btae669_Supplementary_Data [file btae669_supplementary_data.pdf]

Supplementary Material for

RUCova: Removal of Unwanted Covariance in mass cytometry data

Rosario Astaburuaga-García, Thomas Sell, Samet Mutlu, Anja Sieber, Kirsten Lauber, Nils Blüthgen

R package is available on https://github.com/molsysbio/RUCova. Detailed documentation, data, and the code required to reproduce the results are available on https://doi.org/10.5281/zenodo.10913464.

Contents

S1. Surrogates of sources Unwanted Covariance ($\vec{x}_i$) for RUCova 2

S2. RUCova output 2

S3. ASCQ-Ruthenium 6

S4. Experimental details 8

S4.1 Cell culture 8

S4.2 Mass cytometry 8

S5. Apoptotic cells in the HNSCC data set 11

S6. Validation study 12

S7. FACS-sorted data set 13

S1. Surrogates of sources Unwanted Covariance ($\vec{x}_i$) for RUCova

RUCova comprises two major steps. First, it fits a multivariate model for each measured marker (m) across cells (i) from samples (j_i) with respect to the surrogates of sources of unwanted covariance (SUC) $\vec{x}_i$ (Eq. 1). Second, it eliminates such dependency by assigning the residuals ϵ of the model as the new modified expression of the marker (Eq. 5).

Since cell volume and labeling efficiency cannot be directly measured with mass cytometry, we use four Surrogates of sources of Unwanted Covariance (SUCs): (1) Mean DNA: Mean value of normalised iridium channels, (2) Mean BC: Mean value of the highest (used) barcoding isotopes per cell, (3) pan Akt, and (4) total ERK.

$$\vec{x}_i = [\text{mean DNA}_\text{i}, \text{mean highest BC}_\text{i}, \text{total ERK}_\text{i}, \text{pan Akt}_\text{i}] \quad \text{(S1)}$$

Mean DNA per cell i was calculated as:

$$\text{mean DNA}_\text{i} = \frac{\text{Ir191}_\text{norm,i} + \text{Ir193}_\text{norm,i}}{2} \quad \text{(S2)}$$

where Ir191_norm and Ir193_norm are the percentile-normalised intensities (*e.g.,* to the 95th percentile) of the iridium intercalators serving as DNA stains for each cell i. The mean BC signal per cell i was calculated as:

$$\text{mean highest BC}_\text{i} = \frac{1}{\text{N}_\text{BC}} \sum_{\text{k}=1}^{\text{N}_\text{BC}} \text{BC}_\text{i,k,norm} \quad \text{(S3)}$$

where BC_norm are the percentile-normalised intensities of the barcoding isotopes across all cells (*e.g.,* to the 95th percentile), N_BC is the number of barcoding isotopes used per cell (e.g.: $\text{N}_\text{BC} = 3$ for the Fluidigm kit of Palladium isotopes), and BC_k is the barcoding isotope with the k-th highest signal in cell i, meaning the isotope was used in that cell to barcode it. The surrogates pan Akt and total ERK are markers included in our mass cytometry panel.

By taking the zero-centered distributions of the SUCs ($x_i^c = x_i - \frac{1}{N_i}\sum_i^{N_i} x_i$), the mean values of the markers m across all cells are kept after applying RUCova (Fig. 1). If a more conservative approach is desired where the fold changes between samples should be kept, each SUC should be centered per sample. This approach is illustrated in Fig. S1) for the three RUCova models : simple (Eq. 2), offset (Eq. 3), and interaction (Eq. 4). Similarly, when using PCs as the predictive variables, SUCs can be z-score normalised by sample before performing PCA.

Performing PCA on the SUCs may be useful, especially to better identify axes correlating with confounding factors such as PC1 and cell size (Fig 2H).

S2. RUCova output

Here we illustrate the application of RUCova using the Head-and-neck squamous cell carcinoma (HNSCC) data set (from Fig.2,3), for which we choose the interaction model $M_3(\vec{x}_i, j_i)$ (Eq.4). Fig.S2A shows the distribution of the SUCs. PC1 (of a PCA on the SUCs) was not only dependant on one SUC but rather on the combination (loadings in Fig. S2B).

One of the outputs of RUCova is the adjusted R-squared (R^2_adj) or coefficient of determination, which is a useful metric to evaluate the goodness of fit (Fig. S2C). Quantifies the proportion of variance in the dependent variable (marker) explained by the model's independent variables (surrogates). The adjusted R-squared is *adjusted* by the number of independent variables used to predict the target variable. This is done to account for the automatic increase of R^2 values when extra explanatory variables are added to the model. By analysing the R^2_adj,we can determine whether adding new variables increases the model fit.

$$R^2_{adj} = \Big(1 - \underbrace{(1 - \frac{SS_{res}}{SS_{tot}})}_{R^2}\Big) \cdot \frac{n-1}{n-q-1} \quad \text{(S4)}$$

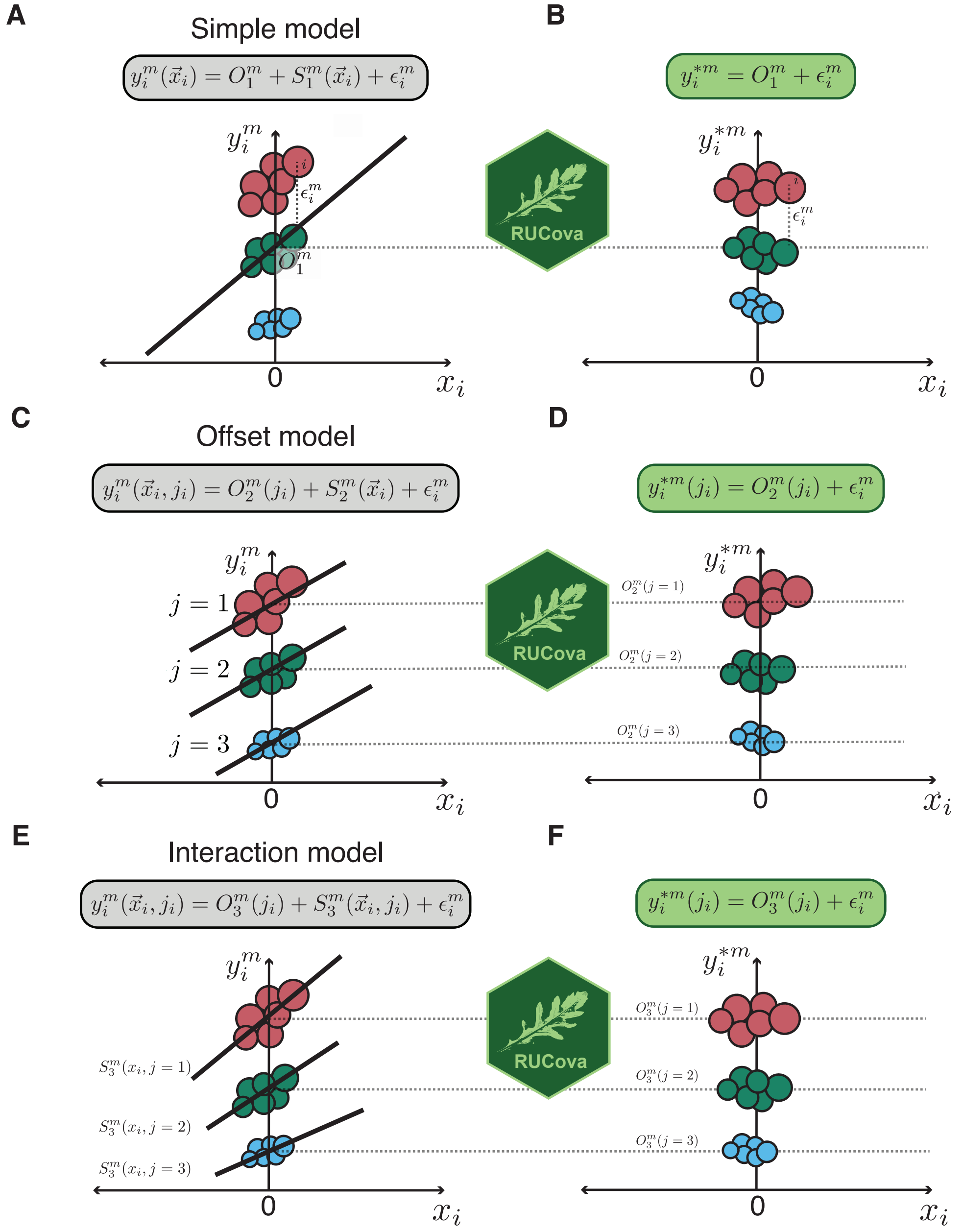

Figure S1: **Illustration of the RUCova method and its three different models, when surrogates are centered per sample. A, C, E)** Original expression y_i^m of a marker m before RUCova as a function of a centered expression of a SUC or PC. Illustrative regression line and equation corresponding to each model. **B, D, F)** Modified expression y_i^{*m} of a marker m after RUCova. **D, F)** Keeping the offset $O^m(j_i)$ between samples j. **A, B)** Simple model: one fit across the input data set with intercept O_1^m and residuals ϵ_i. **C, D)** Offset model: one slope $S_2^m(\vec{x}_i)$ for the whole input data set and different intercepts $O_2^m(j_i)$ between samples. **E, F)** Interaction model: one fit per sample j with intercepts $O_3^m(j_i)$ and slope $S_3^m(\vec{x}_i, j_i)$.

where $SS_{res} = \sum_i \epsilon_i^2$ is the residual sum of squares, ϵ_i are the residuals of the model, $SS_{tot} = \sum_i (y_i - \overline{y})$ is the total sum of squares, $\overline{y}$ is the mean value of the marker, n is the sample size (total number of cells i) and q is the number of explanatory variables in the model.

The model coefficients are also included in the output of RUCova. Assessing the slope coefficients is useful to better understand the effect size of each SUC on the markers. We standardised the slope coefficients in order to make them comparable (Fig. S2D). In the case of the interaction model, where the slopes α^m depend on samples j_i and the SUC p, we standardised the slope by multiplying it by the standard deviation of the corresponding SUC in

each sample j ($\sigma_{x_{j_i,p}}$) and dividing it by the standard deviation of the marker m in sample j ($\sigma_{y^m_{j_i}}$) :

$$\alpha^{m*}_{j_i,x_{i,p}} = \alpha^{m}_{j_i,x_{i,p}} \cdot \frac{\sigma_{x_{j_i,p}}}{\sigma_{y^m_{j_i}}} \tag{S5}$$

Correlation coefficients before and after RUCova are depicted in Fig. S3 and S4.

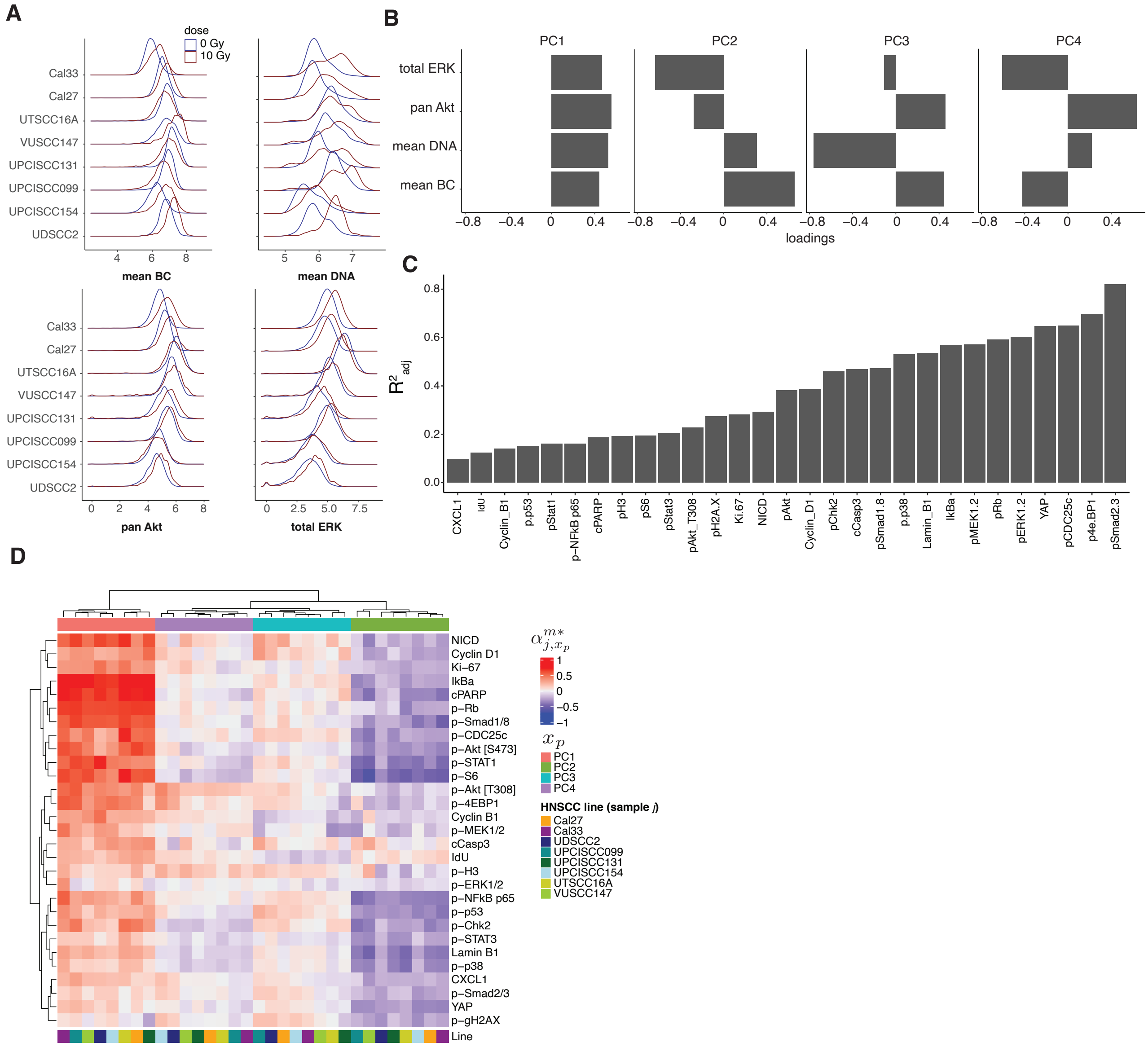

Figure S2: **A)**. Asinh-transformed values of surrogates of sources unwanted covariance (SUC) across cell lines and irradiation conditions. **B)**. Loadings of a PCA based on asinh-transformed and z-score normalised SUCs. **C)**. Adjusted R-squared for each marker after applying the interaction model. **D)**. Standardised slope coefficients for each marker after applying the interaction model with cell lines as samples.

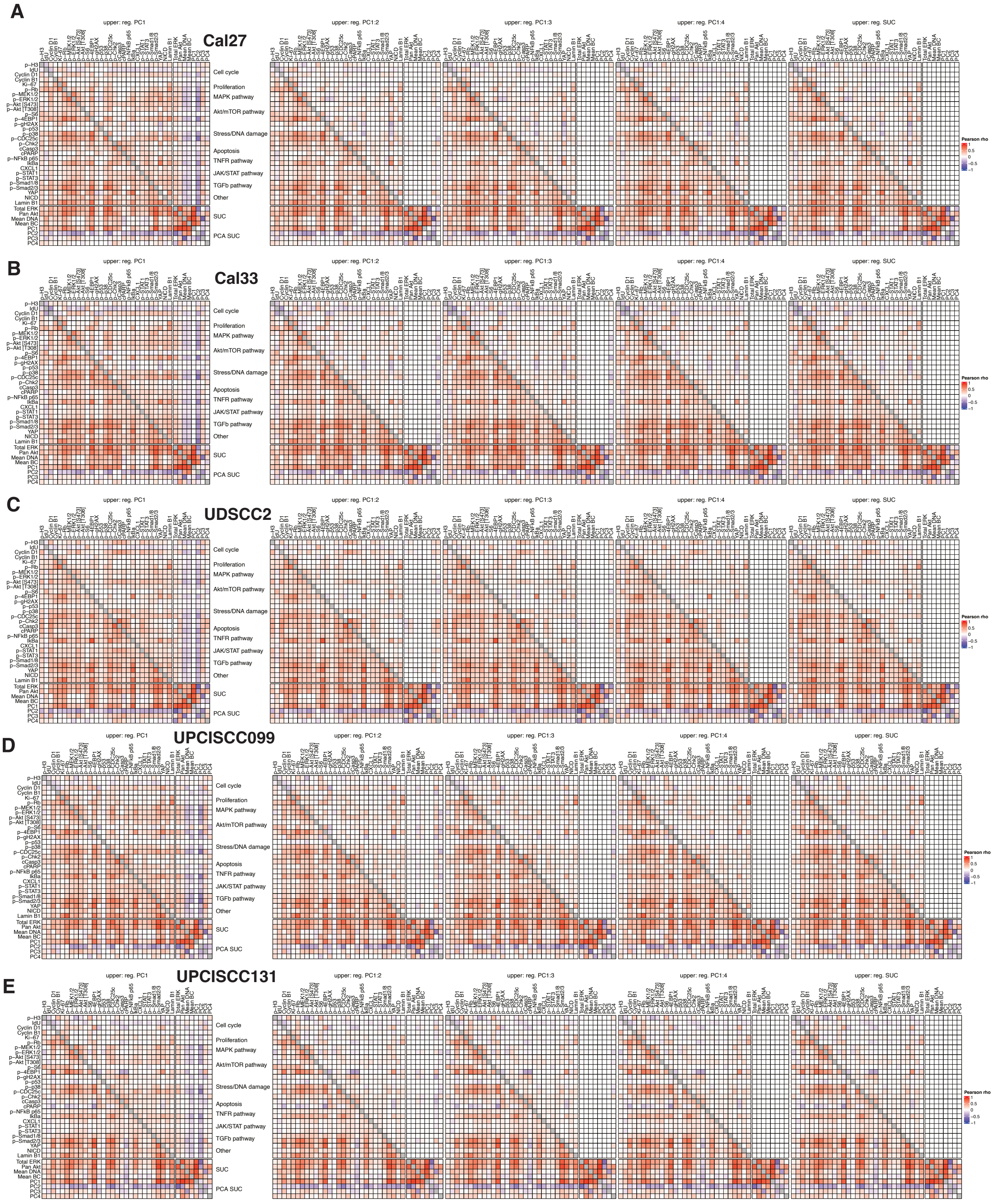

Figure S3: Correlation heatmap with the lower (upper) triangle showing the Pearson correlation coefficients between marker values before (after) RUCova based on PC1 (first) to PC1:4 (fourth) and the four SUCs (fifth), across cells from 0 and 10 Gy in the cell lines **A)** Cal27, **B)** Cal33, **C)** UDSCC2, **D)** UPSCISCC099, **E)** UPSCISCC131.

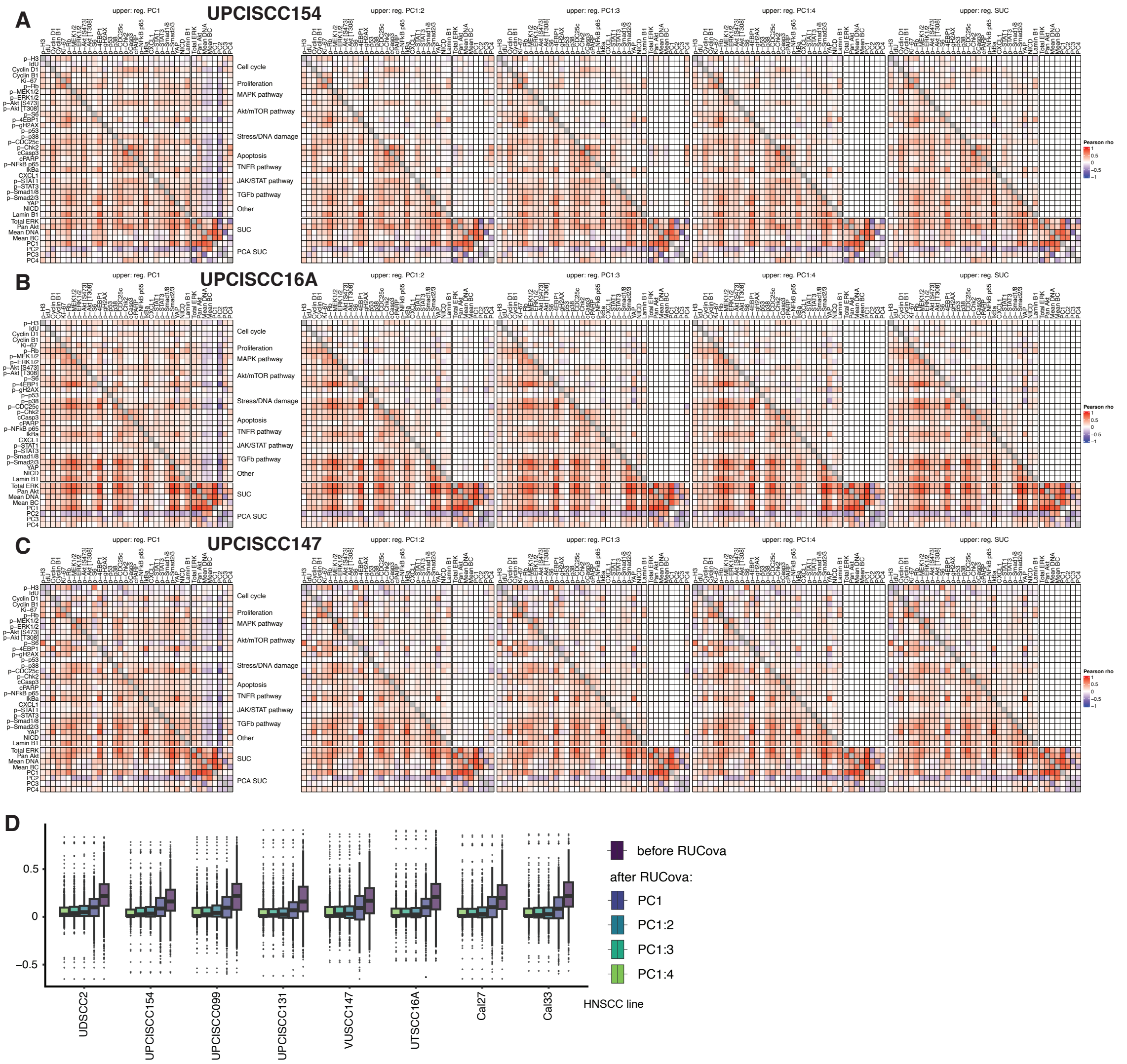

Figure S4: **A,B,C)** Correlation heatmap with the lower (upper) triangle showing the Pearson correlation coefficients between marker values before (after) RUCova based on PC1 (first) to PC1:4 (fourth) and the four SUCs (fifth), across cells from 0 and 10 Gy in the cell lines **A)** UPSCISCC154, **B)** UPSCISCC16A, **C)** UPSCISCC147. **D)** Boxplots for Pearson correlation coefficients between markers per cell line across cells from 0 and 10 Gy, based on the expression values before and after RUCova.

S3. ASCQ-Ruthenium

Other markers may be used as SUCs, for example, the Ruthenium isotopes proposed by Rapsomaniki et al. [2018]). However, they might not add additional information as we observed a high correlation with the mean DNA values (Fig.S5A) in their published data set. They proposed normalising the marker intensity by the mean Ruthenium staining per cell. Consistently, dividing by the mean DNA leads to very similar normalised values (Fig.S5B, first and second heatmap). This approach assumes the same relationship between all markers and cell volume, which might not be correct. Consequently, many correlations between markers and ruthenium or mean DNA are kept after normalisation. On the contrary, by regressing-out the correlations with Ruthenium or DNA (Fig.S5B, third to fifth heatmap) with RUCova and allowing different relationships between cell volume and marker abundance for different markers, all the correlations with ruthenium and DNA stainings were removed.

We tested the Ruthenium compounds in the Cal33 cell line, and computed the PCA in our data set based on the SUCs total ERK, pan Akt, mean Ru, and mean DNA. The variance explained by PC1 was 64% (Fig. S5C) and all SUCs had positive loadings for PC1 (Fig. S5D). As Rapsomaniki et al. [2018] claimed that ruthenium correlates with cell volume, we concluded is not possible to disentangle the staining efficiency from cell size in this data set based

on our SUCs and principal component analysis.

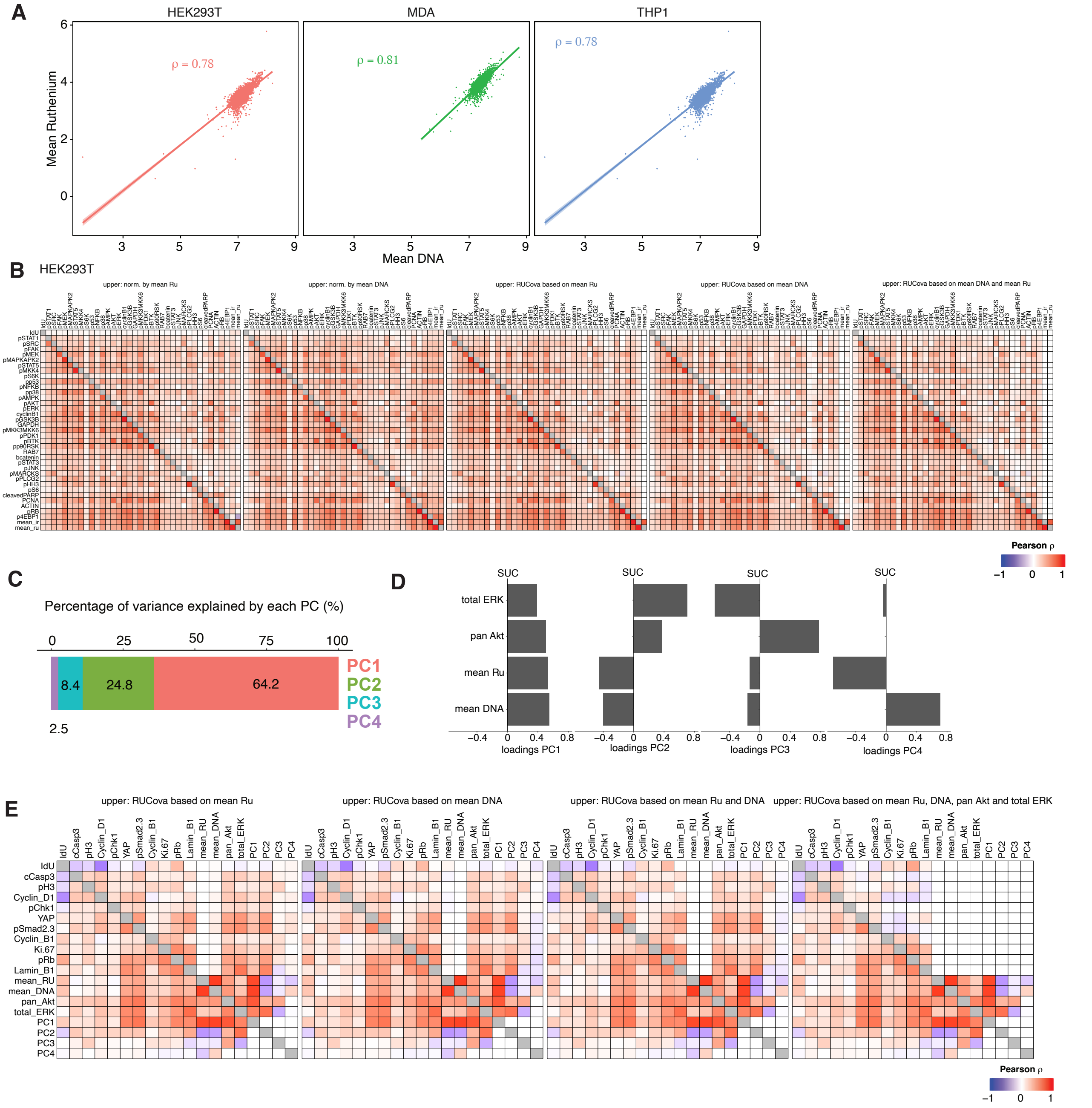

Figure S5: **A)** Scatter plots of asinh-transformed signals of mean Ruthenium and mean DNA staining in three different cell lines (Data from [Rapsomaniki et al., 2018]). **B)** Pearson correlation coefficients between marker abundances in the HEK293T cell line (Data from [Rapsomaniki et al., 2018]) before any normalisation (lower triangle) and after the normalisation or regression indicated on the top of each heatmap. **C)** Variance explained by each PC calculated across the 4 SUCs (total ERK, pan Akt, mean Ru, mean DNA). **D)** Loadings for each PC of a PCA based on the 4 SUCs depicted in the y axis.s **E)** Pearson correlation coefficients between marker abundances in the Cal33 cell line before RUCova (lower triangle) and after RUCova (upper triangle) following based on the SUCs indicated on the top of each heatmap.

S4. Experimental details

S4.1 Cell culture

Cells from 10 HNSCC cell lines (UDSCC2, UPCISCC040, Cal27, UPCISCC099, UPCISCC131, UPCISCC154, UM-SCC1, VUSCC147, UTSCC16A, Cal33) were cultured in DMEM supplemented with 10 % FCS, 1 % Glutamax (Gibco, 35050061), and 1 % Penicillin/Streptomycin (Gibco, 15140122). Cells were seeded into 6-well plates at day -1 (for 0 Gy 400.000 cells/well, for 10 Gy 700.000 cells/ well). Right before radiation treatment at day 0, medium was replaced with fresh medium, and cells were irradiated with 10 Gy using an RS225 X-ray cabinet (X-Strahl, Camberley, UK) operated at 200 kV/10 mA (Thoraeus filter, 1 Gy in 63 s), or left untreated. At 48 hours, the cells were prepared for fixation as follows: 30 min prior to fixation, IdU was added to the cell culture medium for a final concentration of 10 μM (1:5000 from stock solution; Standard Biotools, 201127), plates were rocked well but gently, and incubated at 37 °C for 30 min. Culture medium was discarded and plates washed once with PBS. Cell-ID™ Cisplatin (2:1000 from stock, 2 μM in PBS; Standard Biotools, 201064) was added to cells for 5 min at 37 °C and cells were washed once with culture medium (full medium including FCS) followed by 1x PBS washing. Fixation of cells was performed using methanol-free formaldehyde (2 % in PBS; stock from Pierce™ 16 %, 28906) at 37 °C for 15 minutes. Reaction was stopped by adding protein-containing medium followed by 2x PBS washing. Subsequently, cells were dissociated by adding Accutase for 45 min at 37 °C, scraping, pipetting, and straining through Flowmi (40 μm, BAH 136800040, Sigma) filter. Cells were transferred into low-binding reaction tube. After spinning cells down at 800 g for 5 min and discarding the supernatant, cells were re-suspended in 500 μl PBS/BSA (10 %) + 10 % DMSO and stored at −20 °C.

For cell size determination, same procedure was followed (without IdU/Cisplatin administration) including fixation and washing steps. Then, images were acquired through 6-well plate using Zeiss AxioObserver Z1 inverted microscope (Fig. S6A). The acquired images were then analyzed for cell area determination using ZEN 2.3 software and embedded toolkit. A minimum of 12 cells were marked for each condition (Fig. S6B). After pre-processing of mass cytometry data, two cell lines (UMSCC1 and UPCISCC040) were excluded from the analysis due to low number of cells in the irradiated condition ($n < 500$, Fig. S6C).

For FACS sorting and ASCQ-Ru experiments, similar cell culture procedures were followed using only Cal33 cells, but plates were not irradiated. For the FACS sorting experiment, cells were perturbed in the following way: 24 h prior fixation, FCS was removed from the growth medium for all samples but one control. Perturbations were either Gefitinib for 24 h at 10 μM, EGF for 30 min at 25 ng/ml, IFN-β for 30 min at 25 ng/ml, Etoposide for 2 h at 42 μM, GDC0941 for 24 h at 1 μM, or IGF for 30 min at 100 ng/ml. IdU incubation was started in parallel as described above.

S4.2 Mass cytometry

Frozen samples were thawed at 37 °C and washed 1x in PBS. Free nucleic acids were digested by incubating each sample with Benzonase (Pierce, 88700; 100 U/ml in PBS). The Cell ID™ 20-Plex Pd Barcoding Kit (Standard Biotools, 201060) was used for sample barcoding according to manufacturer's instructions. Subsequent processing steps were performed on multiplexed cell pools. For experiments with more than 20 conditions to be barcoded, monoisotopic Cisplatin was used as an additional identifying factor of cells already pooled based on Pd barcodes. Multiplexed cells were permeabilised by chilling for 10 min followed by incubating with ice-cold methanol for 15 minutes. After 2x washes in Cell Staining Buffer (Standard Biotools, 201068), mass-tagged antibodies were added for 30 min at room temperature, followed by 2 more washes in Cell Staining Buffer. Iridium DNA intercalator (Standard Biotools, 201192A) was added at a concentration of 63 μM in PBS for 20 min at room temperature. Subsequently, the sample was washed once in PBS and stored overnight at 4 °C in methanol-free formaldehyde (2 % in PBS; stock from Pierce™ 16 %, 28906). On the following day some samples were treated with 25 μg/ml ASCQ-Ru in 0.1 M $NaHCO_3$. Other samples were divided into "small" and "large" cells via FACS sorting based on FSC-A/SSC-A ratios. All samples were washed 2x in doubly distilled water and filtered through a 10 μm cell strainer prior to acquisition in a CyTOF 2 (Helios upgrade) mass cytometer.

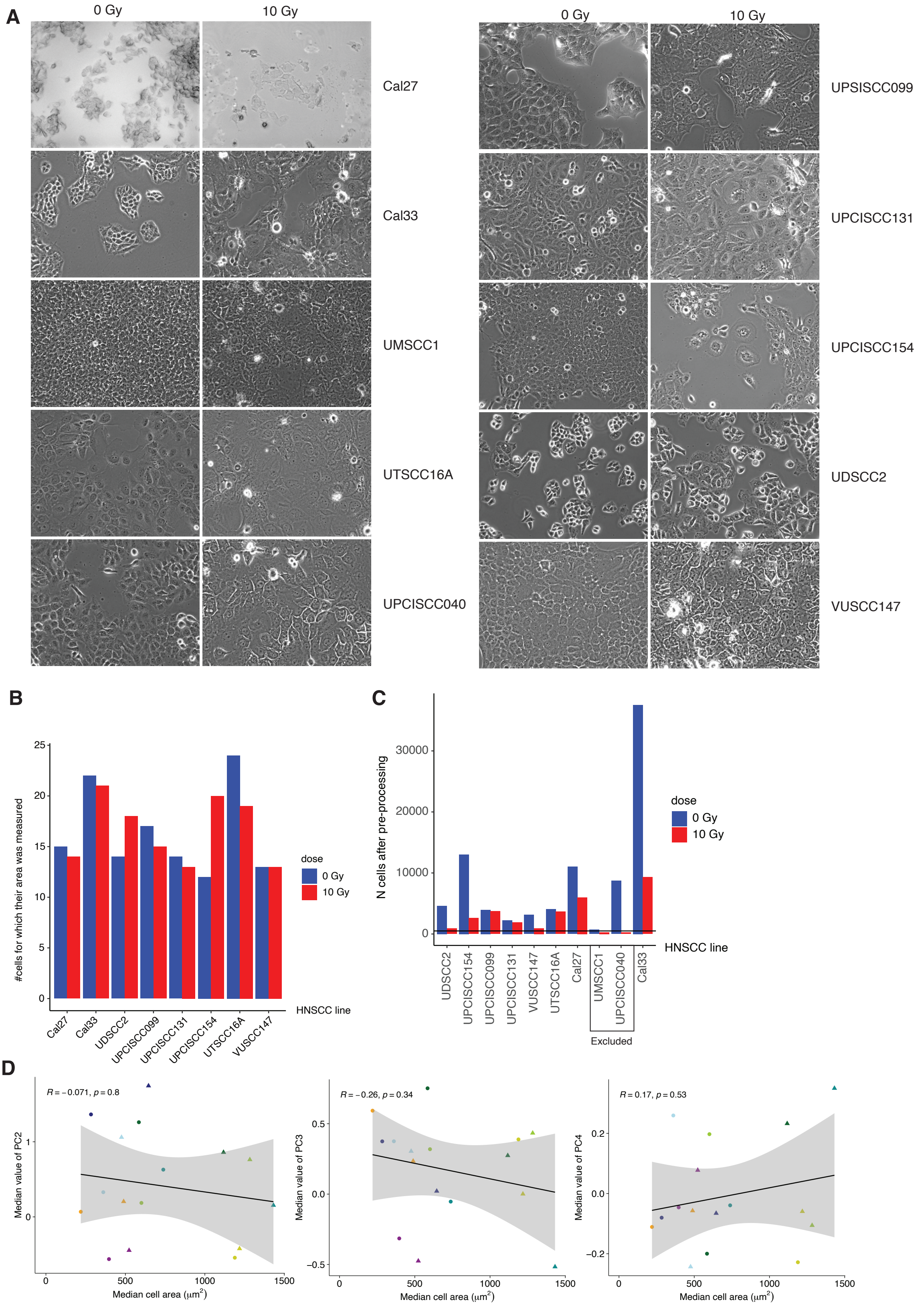

Figure S6: **A)** Microscopy images for the 10 HNSCC lines in each irradiated condition. **B)** Number of cells per cell line and condition for which the cell area was quantified. **C)** Number of cells per cell line and condition in the mass cytometry data set after pre-processing. UMSCC1 and UPCISCC040 were excluded due to low cell number ($n < 500$, horizontal line **D)** Scatter plots of measured mean cell area and mean value of PC2 (left), PC3 (middle), and PC4 (right).

Table S1: Antibody panel for the three data sets: HNSCC, FACS-sorted and ASCQ-Ru

label	Category	target	HGNC symbol	vendor	clone	HNSCC data set	FACS-sorted data set	ASCQ-Ru data set
141Pr	Stress/DNA damage	pChk2 [T68]	CHEK2	CST	C13C1	X	X	
142Nd	Apoptosis	cCasp3	CASP3	Fluidigm	D3E9	X	X	X
143Nd	Apoptosis	cPARP	PARP1	Fluidigm	F21-852	X	X	
144Nd	SUC	pan Akt	AKT1	CST	40D4	X	X	X
145Nd	Cell cycle	p-H3 [S28]	H3-4	BioLegend	HTA28	X	X	X
146Nd	Cell cycle	Cyclin D1	CCND1	Abcam	SP4	X	X	X
147Sm	Stress/DNA damage	p-H2AX [S139]	H2AFX	Fluidigm	JBW301	X	X	
148Nd	Stress/DNA damage	p-Chk1 [S345]	CHEK1	CST	133D3			X
149Sm	TGFb pathway	p-Smad1 [S463/S465] / p-Smad8 [S465/S467]	SMAD1, SMAD9	BD	N6-1233	X	X	
150Nd	Other	YAP	YY1AP1	CST	D8H1X	X	X	X
151Eu	MAPK pathway	p-MEK1/2 [S217/221]	MAP2K1, MAP2K2	CST	41G9	X	X	
152Sm	Akt/mTOR pathway	p-Akt [S473]	AKT1	Fluidigm	D9E	X	X	
153Eu	TGFb pathway	p-Smad2 [S465/467] / p-Smad3 [S423/425]	SMAD2, SMAD3	CST	D27F4	X	X	X
154Sm	JAK/STAT pathway	p-Stat1 [T701]	STAT1	BioLegend	A17012A	X	X	
155Gd	TNFR pathway	p-NF-kB p65 [S536]	RELA	CST	93H1	X	X	
156Gd	Stress/DNA damage	p-p38 [T180/Y182]	MAPK14	Fluidigm	D3F9	X	X	
158Gd	JAK/STAT pathway	p-Stat3 [Y705]	STAT3	Fluidigm	4/P-STAT3	X	X	
159Tb	Stress/DNA damage	p-CDC25c [S216]	CDC25C	CST	63F9	X	X	
160Gd	Cell cycle	Cyclin B1	CCNB1	BD	-11 GNS	X	X	X
162Dy	Proliferation	Ki-67	MKI67	Fluidigm	B56	X	X	X
163Dy	Proliferation	p-RB [S807/S811]	RB1	BD	J112-906	X	X	X
164Dy	TNFR pathway	IκBα	NFKBIA	Fluidigm	L35A5	X	X	
166Er	JAK/STAT pathway	CXCL1	CXCL1	R&D	20326	X	X	
168Er	Akt/mTOR pathway	p-Akt [T308]	AKT1	CST	D25E6	X	X	
169Tm	Other	GDF15	GDF15	Abcam	EPR19939	X		
170Er	Akt/mTOR pathway	p-4EBP1 [T37/46]	EIF4EBP1	CST	236B4	X	X	
171Yb	MAPK pathway	pERK1/2 [T202/Y204]	MAPK3, MAPK1	Fluidigm	D13.14.4E	X	X	
172Yb	Stress/DNA damage	p-p53 [S15]	TP53	CST	16G8	X	X	
173Yb	Other	NICD	NOTCH1	Abcam	ab8925	X		
174Yb	SUC	total ERK1/2	MAPK3, MAPK1	CST	L34F12	X	X	X
175Lu	Akt/mTOR pathway	p-S6 [S235/236]	RPS6	Fluidigm	N7-548	X	X	
176Yb	Other	Lamin B1	LMNB1	Abcam	EPR22165-121	X		X

S5. Apoptotic cells in the HNSCC data set

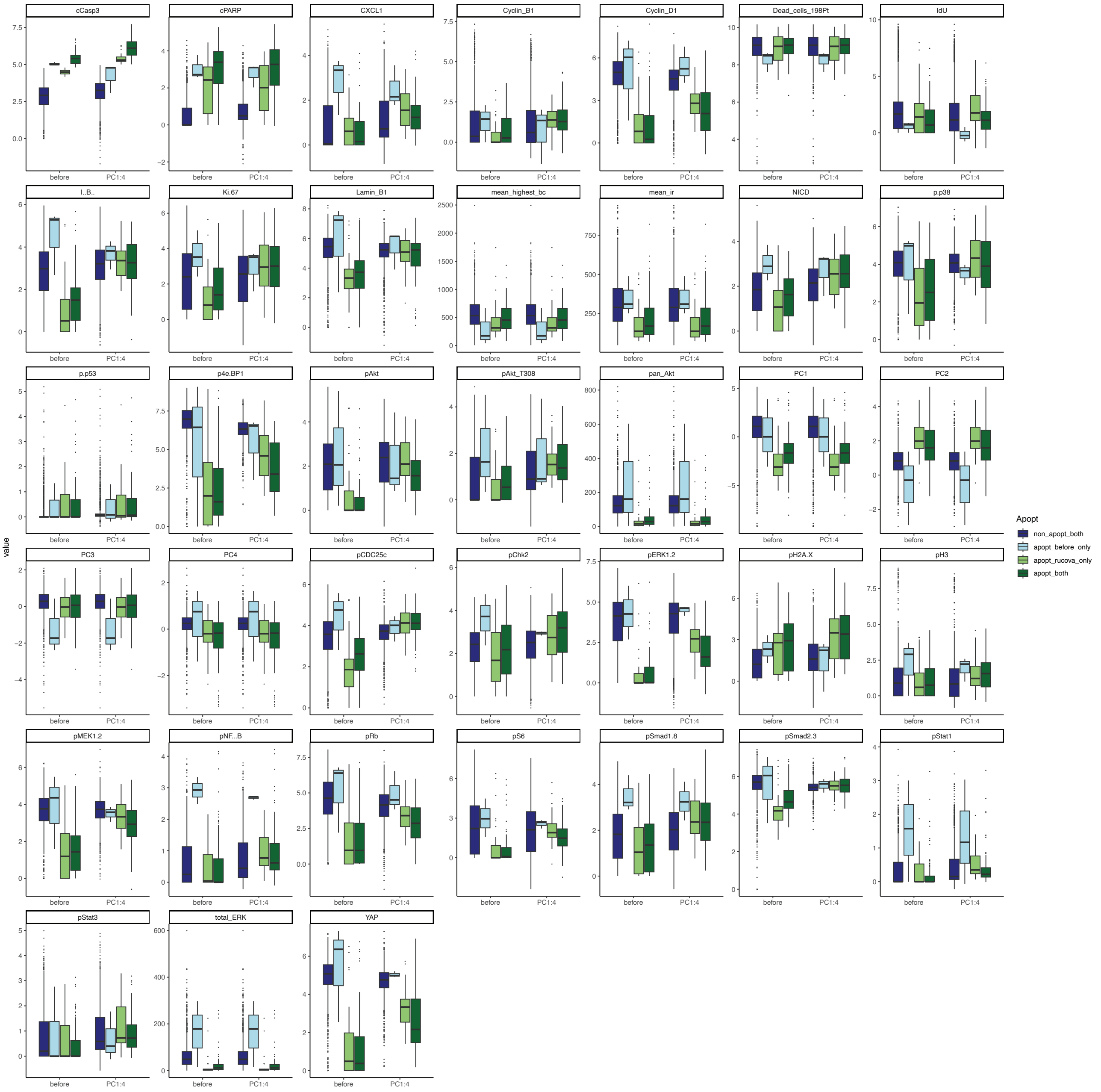

Figure S7: Boxplots of asinh-transformed marker expression irradiated UPCISCC131 cells according to their apoptotic status.

S6. Validation study

We conducted a validation study using the CytoGLMM R package (Seiler et al. [2021]), where we generated 100 simulated mass cytometry datasets, each consisting of 10 markers measured across 10 donors and 2 conditions (treatment and control). For each simulation, we introduced artefacts to test RUCova's ability to recover ground truth correlations. The artefacts were modeled in two ways: (1) Linear artefacts – we multiplied the marker values by the cell size. (2) Non-linear (quadratic) artefacts – we multiplied the marker values by the squared cell size. Cell size values were drawn from a log-normal distribution (mean = 3), and we varied the standard deviation (SD) from 0.5 to 3.5 in increments of 0.5 to simulate different strengths of the artefacts. We then applied RUCova using a simple model with cell size as an explanatory variable.

To assess its performance, we calculated the absolute difference between the markers' Pearson correlation coefficients in the ground truth data and the confounded or regressed datasets (Fig. S8A-B). The results show that RUCova effectively recovers the original correlations across a wide range of artefact strengths, with minimal deviation when the noise is quadratic. This slight deviation is expected due to the non-linear nature of the artefact, but RUCova is able to handle even non-linear noise like exponential artefacts, as it operates on log- or asinh-transformed values, making exponential noise multiplicative. Additionally, we leveraged CytoGLMM's feature to identify significantly different markers between conditions across donors. This allowed us to evaluate RUCova's performance in recovering the correct expression patterns. The task was to predict markers that showed significant differences between treatment and control conditions (adjusted p-value < 0.1). As shown in Fig. S8C-D, RUCova achieved precision and recall values close to 1.0 in the regressed dataset, demonstrating its ability to restore meaningful biological signals even in the presence of confounding artefacts.

In summary, this systematic analysis shows that RUCova is highly effective in reconstructing the original correlations and condition-specific patterns, even when varying the type and strength of artefacts.

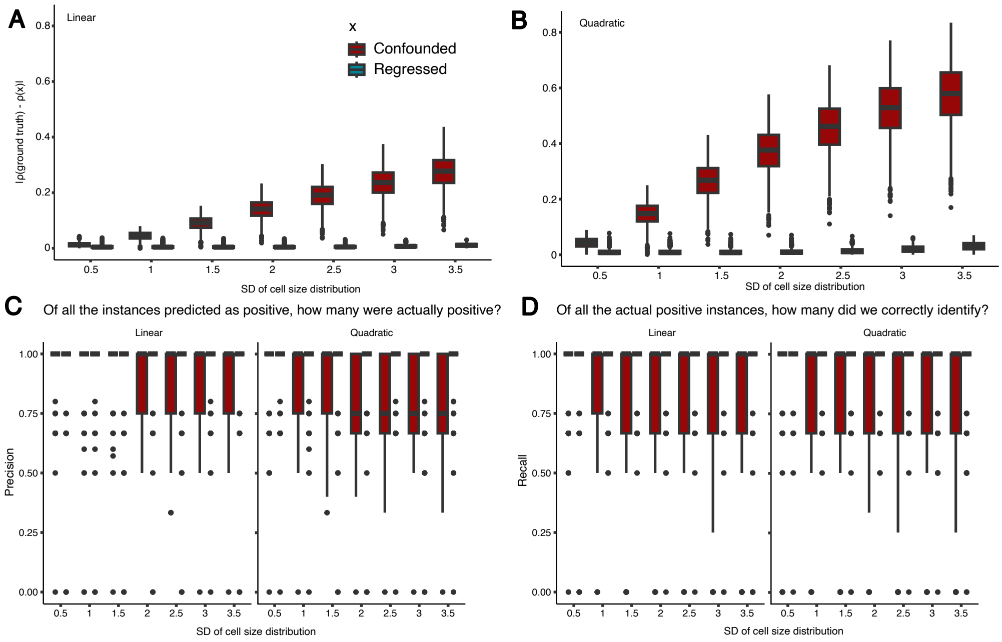

Figure S8: **Validation study. A,B)** Absolute difference in the marker's Pearson correlation coefficient in the simulated ground truth data set versus confounded (dark red) or regressed data after applying RUCova (dark cyan). Different standard deviation (SD) values were used for the log-normal distribution for the confounding cell size. **A)** Linear confounding factor (marker·size). **B)** Quadratic confounding factor (marker·size ^{2}). **C)** Precision and **D)** Recall for classification of significantly different markers between treatment and control.

S7. FACS-sorted data set

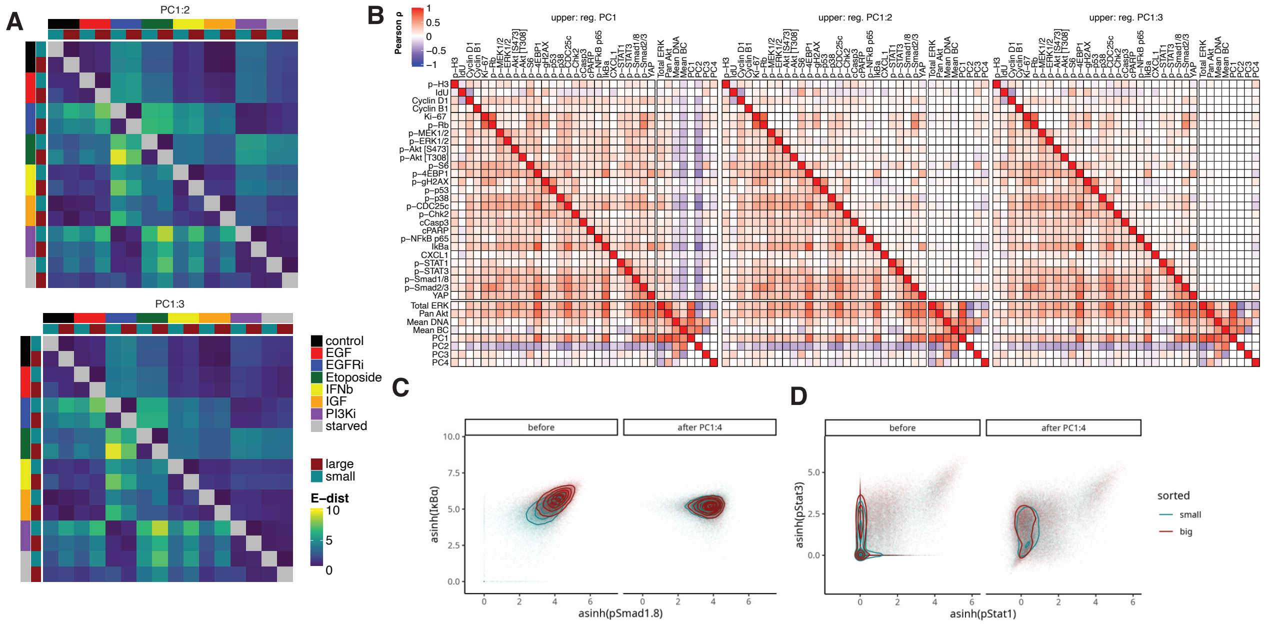

Figure S9: **A)** E-distance heatmap between conditions and sorted populations for data after RUCova using PC1 to PC2 (top) and PC1 to PC3 (bottom). **B)** Correlation heatmap with the upper triangle showing the Pearson correlation coefficients between marker values across all pertubations and sorted populations after RUCova based on PC1 (left), PC1 to PC2 (middle) and PC1 to PC3 (right). **C,D)** Scatter plots of Cal33 cells coloured by sorted population (small and large cells), before (left) and after (right) RUCova based on all four PCs. **C)** Asinh-transformed signals of p-Smad1/8 and IκBα. **D)** Asinh-transformed signals of p-Stat1 and p-Stat3.

References

M. A. Rapsomaniki et al. CellCycleTRACER accounts for cell cycle and volume in mass cytometry data. *Nat Commun*, .

C. Seiler et al. Cytoglmm: conditional differential analysis for flow and mass cytometry experiments. *BMC Bioinformatics*, Mar. 2021. .
